# Supplementary material for: A Humanized Yeast Phenomic Model of Deoxycytidine Kinase to Predict Genetic Buffering of Nucleoside Analog Cytotoxicity
Source: Genes (Basel). 2019 Sep 30;10(10):770. doi: 10.3390/genes10100770 (PMC6826991; doi:10.3390/genes10100770)
Supplement: Supplementary file 1 [file genes-10-00770-s001.zip › Additional_Files/Additional_File7_GOTermHeatmaps.zip!/Additional_File7_GOTermHeatmaps/vacuolar_transport.pdf]

vacuolar transport

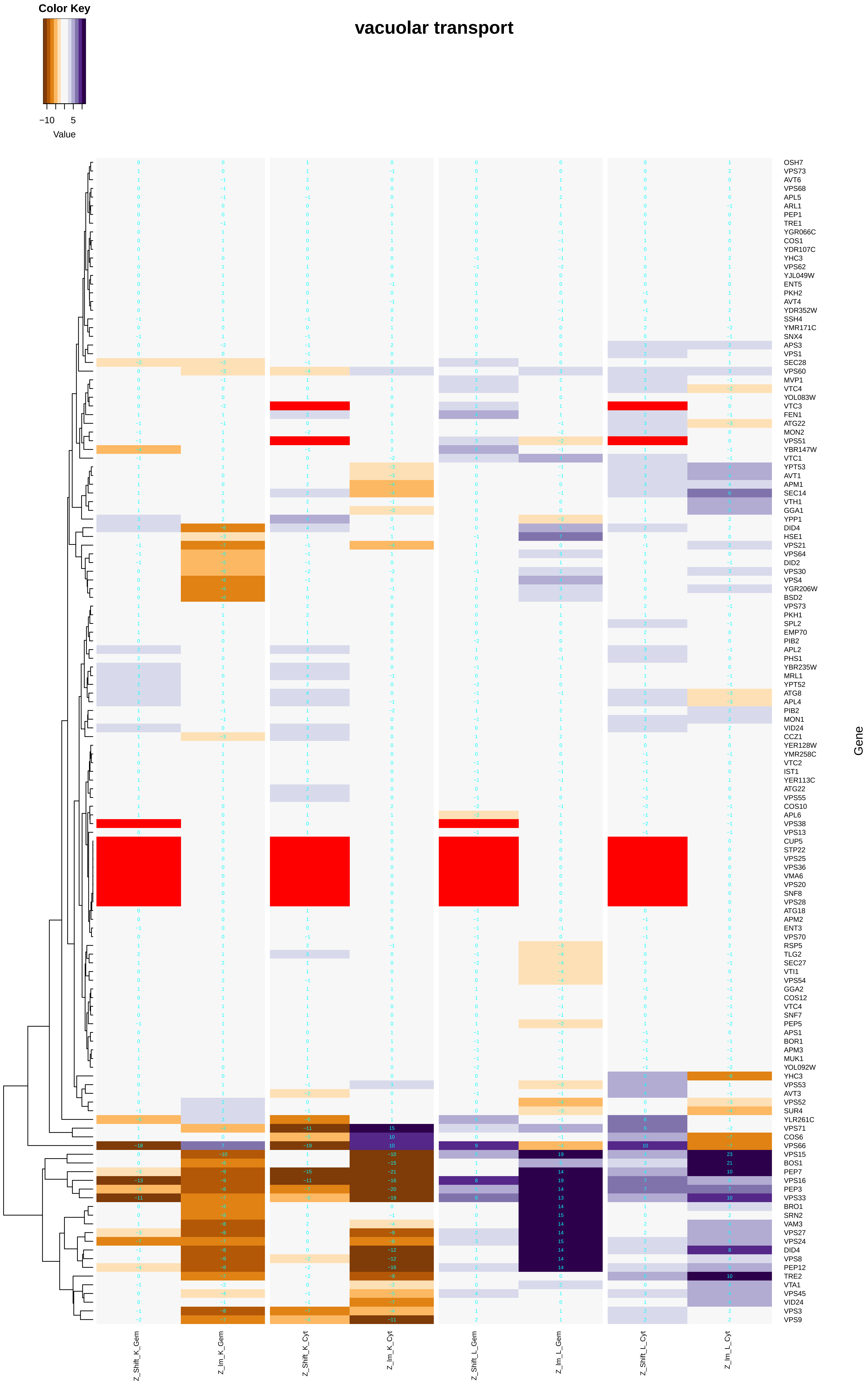

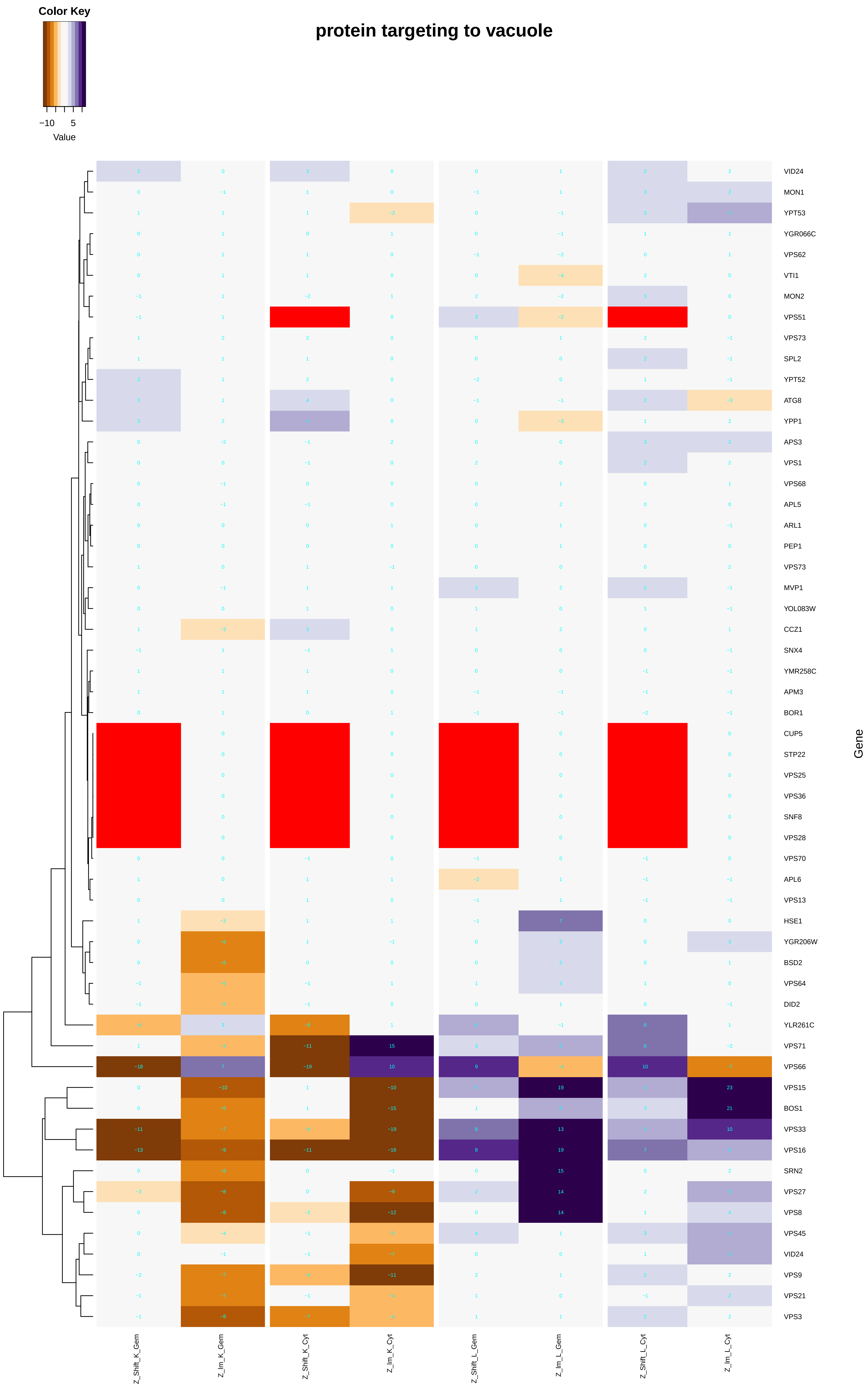
protein targeting to vacuole
Color Key
−10
5
Value
VID24
MON1
YPT53
YGR066C
VPS62
VTI1
MON2
VPS51
VPS73
SPL2
YPT52
ATG8
YPP1
APS3
VPS1
VPS68
APL5
ARL1
PEP1
VPS73
MVP1
YOL083W
CCZ1
SNX4
YMR258C
APM3
BOR1
CUP5
STP22
VPS25
VPS36
SNF8
VPS28
VPS70
APL6
VPS13
HSE1
YGR206W
BSD2
VPS64
DID2
YLR261C
VPS71
VPS66
VPS15
BOS1
VPS33
VPS16
SRN2
VPS27
VPS8
VPS45
VID24
VPS9
VPS21
VPS3
Gene
Z_Shift_K_Gem
Z_Im_K_Gem
Z_Shift_K_Cyt
Z_Im_K_Cyt
Z_Shift_L_Gem
Z_Im_L_Gem
Z_Shift_L_Cyt
Z_Im_L_Cyt

Golgi to vacuole transport

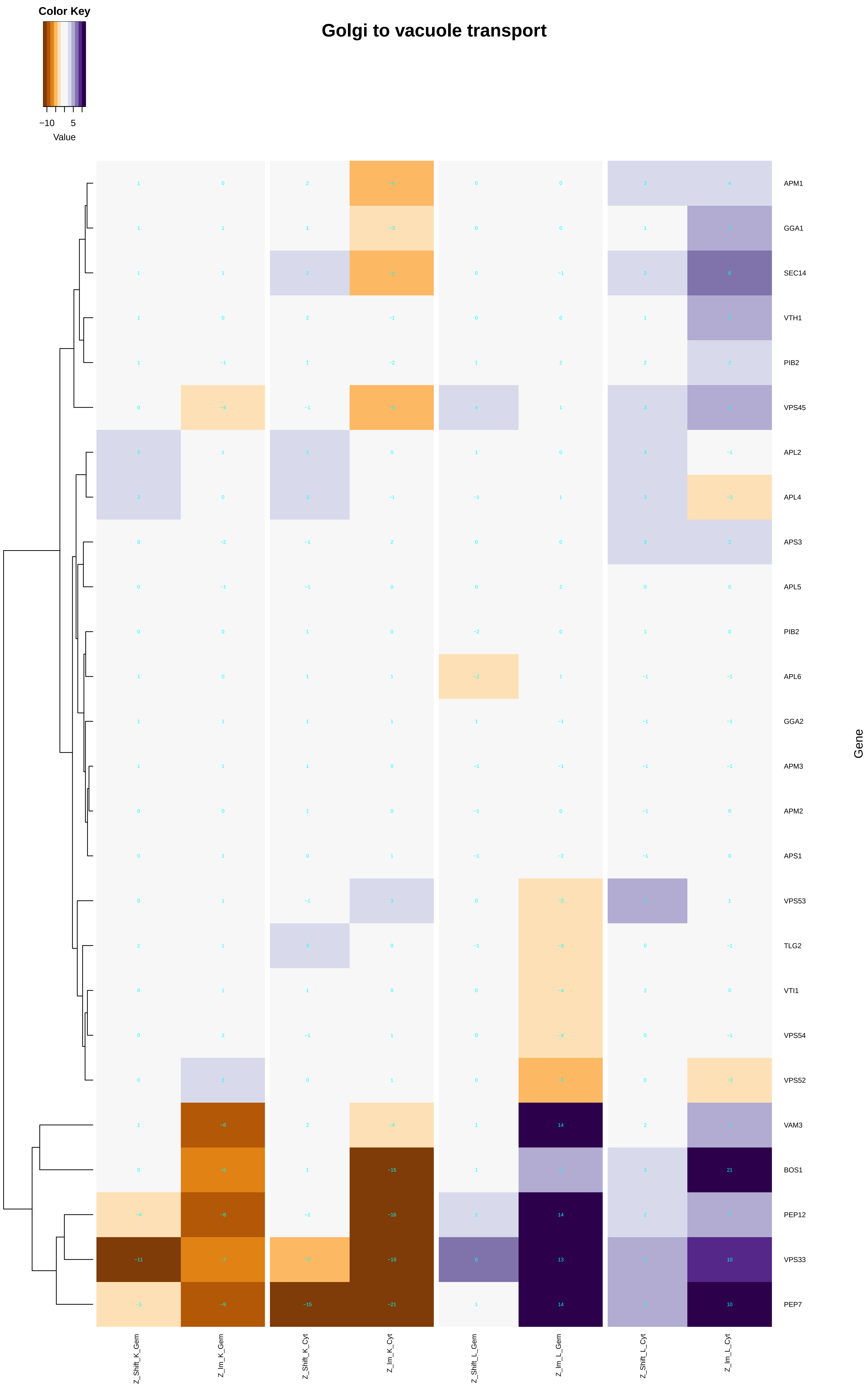

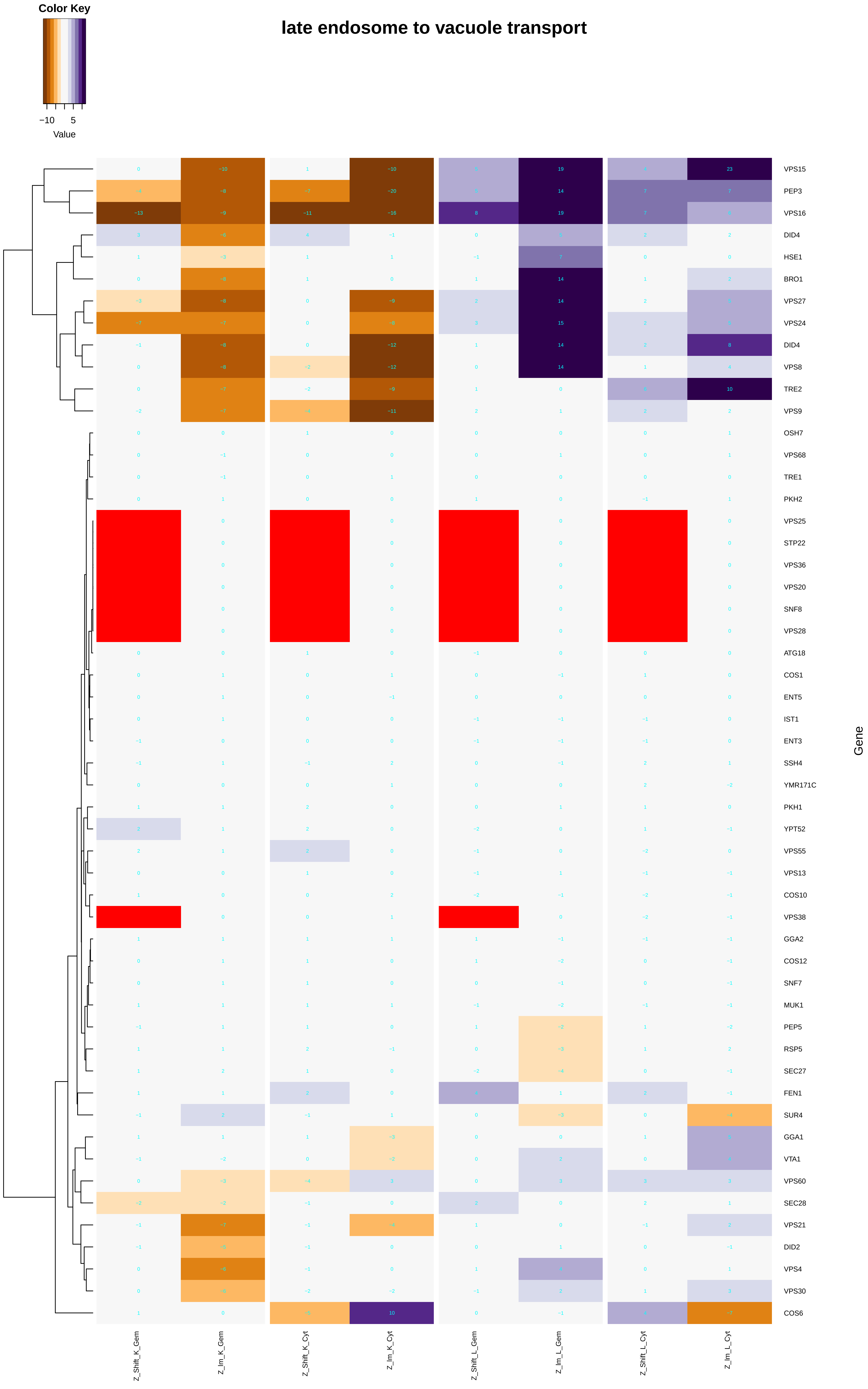
Color Key
−10
5
Value
late endosome to vacuole transport
VPS15
PEP3
VPS16
DID4
HSE1
BRO1
VPS27
VPS24
DID4
VPS8
TRE2
VPS9
OSH7
VPS68
TRE1
PKH2
VPS25
STP22
VPS36
VPS20
SNF8
VPS28
ATG18
COS1
ENT5
IST1
ENT3
SSH4
YMR171C
PKH1
YPT52
VPS55
VPS13
COS10
VPS38
GGA2
COS12
SNF7
MUK1
PEP5
RSP5
SEC27
FEN1
SUR4
GGA1
VTA1
VPS60
SEC28
VPS21
DID2
VPS4
VPS30
COS6
Gene
Z_Shift_K_Gem
Z_Im_K_Gem
Z_Shift_K_Cyt
Z_Im_K_Cyt
Z_Shift_L_Gem
Z_Im_L_Gem
Z_Shift_L_Cyt
Z_Im_L_Cyt

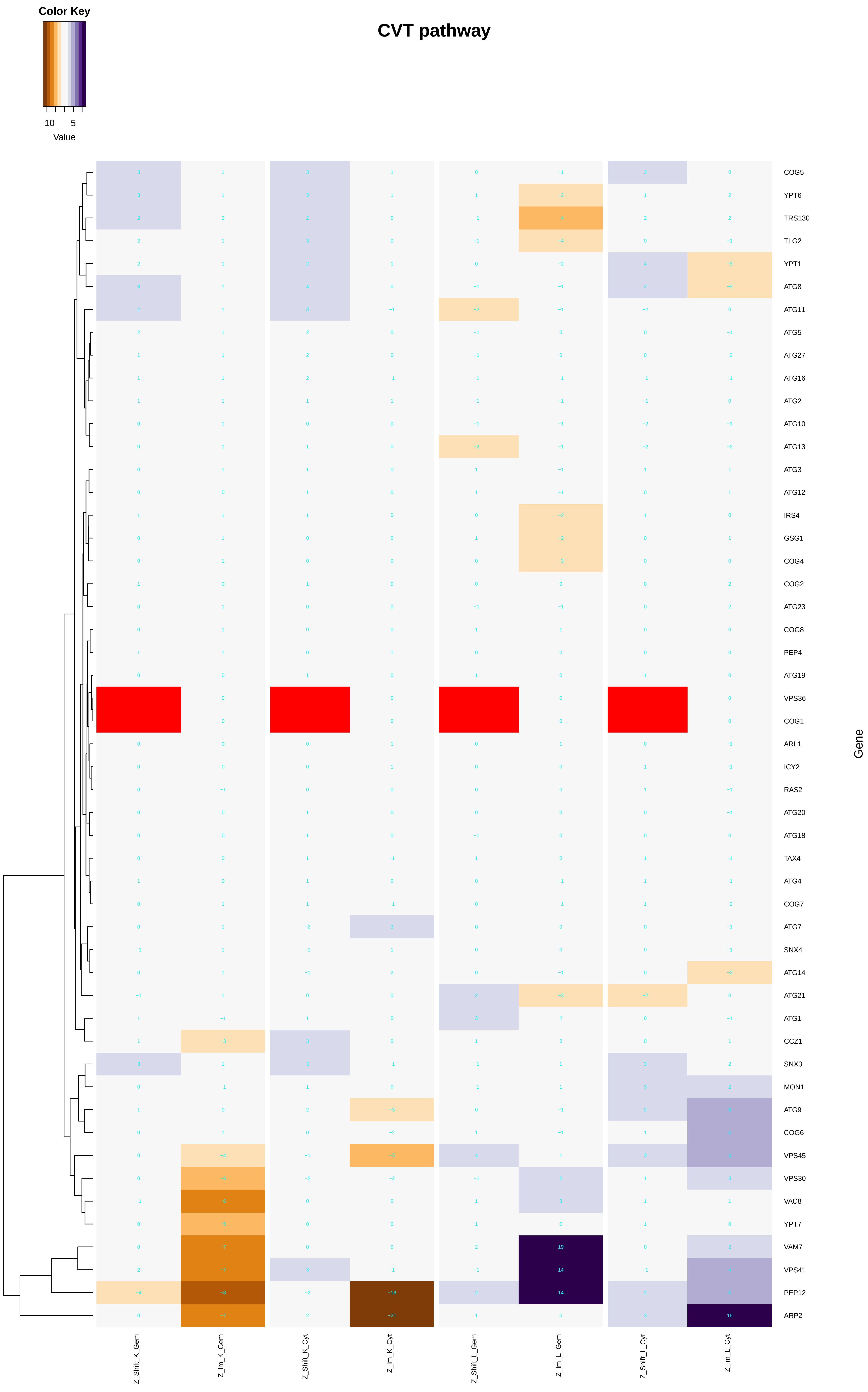
CVT pathway
Color Key
−10
5
Value
Gene
COG5
YPT6
TRS130
TLG2
YPT1
ATG8
ATG11
ATG5
ATG27
ATG16
ATG2
ATG10
ATG13
ATG3
ATG12
IRS4
GSG1
COG4
COG2
ATG23
COG8
PEP4
ATG19
VPS36
COG1
ARL1
ICY2
RAS2
ATG20
ATG18
TAX4
ATG4
COG7
ATG7
SNX4
ATG14
ATG21
ATG1
CCZ1
SNX3
MON1
ATG9
COG6
VPS45
VPS30
VAC8
YPT7
VAM7
VPS41
PEP12
ARP2
Z_Shift_K_Gem
Z_Im_K_Gem
Z_Shift_K_Cyt
Z_Im_K_Cyt
Z_Shift_L_Gem
Z_Im_L_Gem
Z_Shift_L_Cyt
Z_Im_L_Cyt

le involved in ubiquitin−dependent protein catabolic process via the multivesicular body s

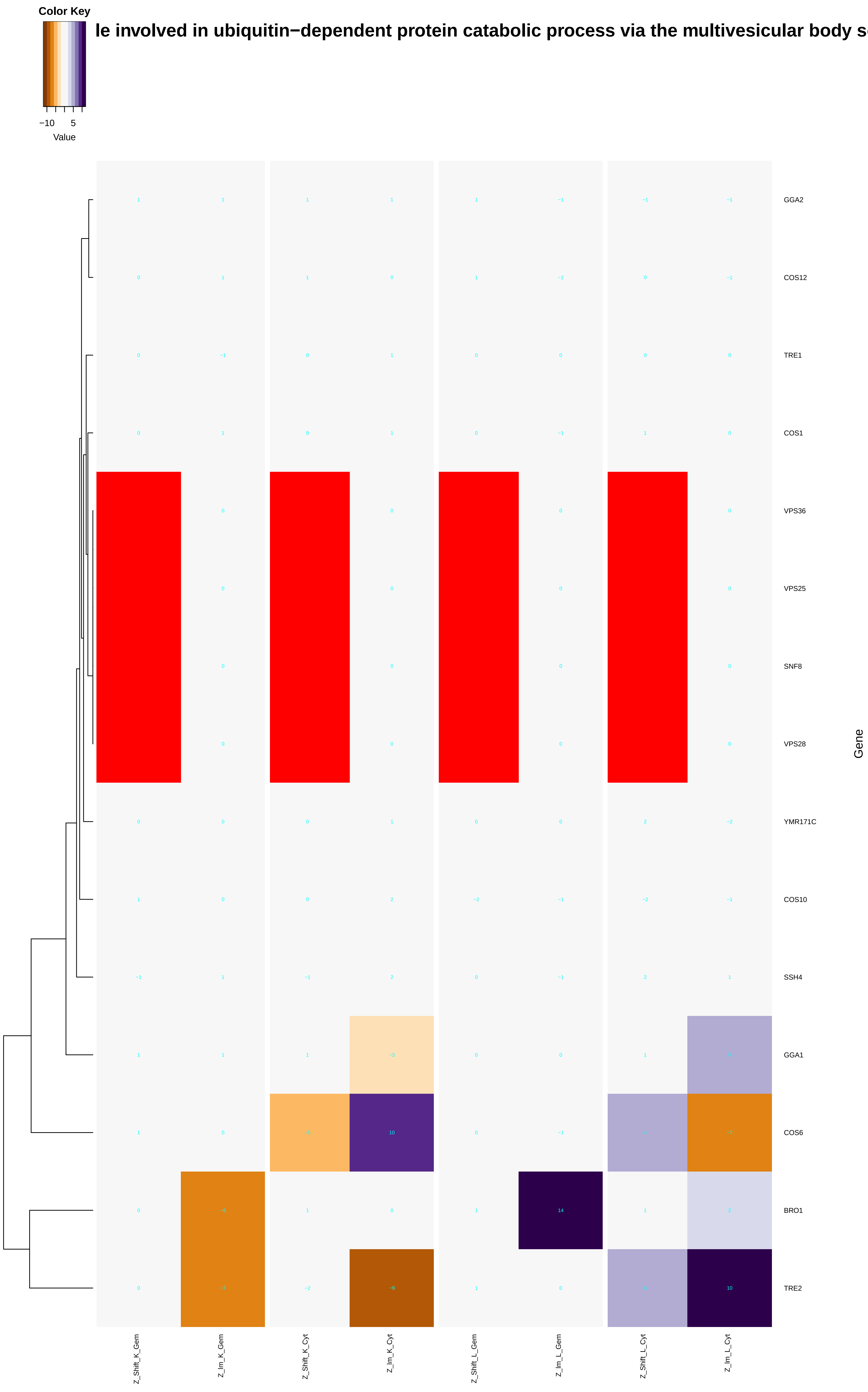

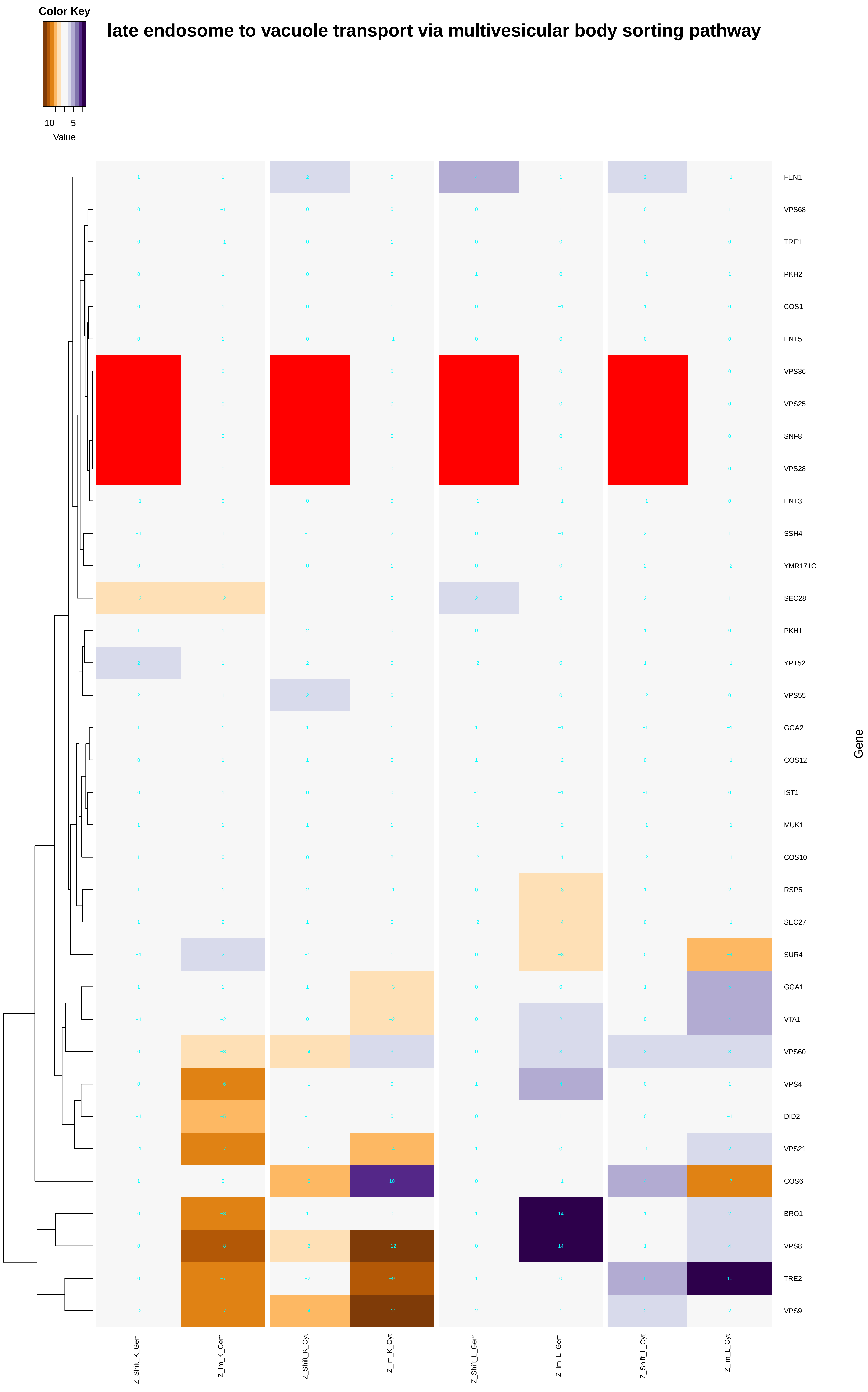

Color Key
−10
5
Value
late endosome to vacuole transport via multivesicular body sorting pathway
FEN1
VPS68
TRE1
PKH2
COS1
ENT5
VPS36
VPS25
SNF8
VPS28
ENT3
SSH4
YMR171C
SEC28
PKH1
YPT52
VPS55
GGA2
COS12
IST1
MUK1
COS10
RSP5
SEC27
SUR4
GGA1
VTA1
VPS60
VPS4
DID2
VPS21
COS6
BRO1
VPS8
TRE2
VPS9
Gene
Z_Shift_K_Gem
Z_Im_K_Gem
Z_Shift_K_Cyt
Z_Im_K_Cyt
Z_Shift_L_Gem
Z_Im_L_Gem
Z_Shift_L_Cyt
Z_Im_L_Cyt
